# The Role of Community Support and Social Networks Among Marginalized Groups in Later Life

**DOI:** 10.1093/geroni/igab046.151

**Published:** 2021-12-17

**Authors:** Samuel Van Vleet

**Affiliations:** Miami University, Oxford, Ohio, United States

## Abstract

As the aging population in the United States continues to grow, so does the need for advancement and critical research to better understand later life experiences. The presence of cumulative disadvantages among racial minorities can often lead to later life health disparities. The goal of this study is to assess the role that social networks and community support play in later life health for marginalized communities. Data from the National Health and Aging Trends Study were analyzed using general linear regression techniques. This allowed for better understanding into the relationships between community support, social networks, race/ethnicity and self-reported health. The final sample included 3,857 participants aged 65 and older. After controlling for other variables, community support and social networks had statistically significant positive relationship with later life health. Race/ethnicity was the strongest predictor of worse later life health. The results of this study show the importance of later life social support for predicting health scores. White participants not only maintained higher health scores as compared to Black and Hispanic participants, but they also reported higher levels of social networks and community support. Findings from this study help build upon the literature regarding community support and social networks in later life.

